# The safety and immunogenicity of a two-dose schedule of CoronaVac, and the immune persistence of vaccination for six months, in people living with HIV: A multicenter prospective cohort study

**DOI:** 10.3389/fimmu.2023.1129651

**Published:** 2023-03-13

**Authors:** Yuxiao Wang, Ying Qiao, Yuqi Huo, Li Wang, Shijie Liang, Maohe Yu, Xinquan Lan, Moxin Song, Xiangjun Zhang, Ying Yan, Junjie Xu

**Affiliations:** ^1^ Clinical Research Academy, Peking University Shenzhen Hospital, Shenzhen Peking University-The Hong Kong University of Science and Technology Medical Center, Shenzhen, China; ^2^ Department of infection, The Second Hospital of Huhhot, Huhhot, China; ^3^ Translational Medicine Research Center, The Sixth People’s Hospital of Zhengzhou, Zhengzhou, China; ^4^ Department of Infection, Heilongjiang Provincial Hospital, Heilongjiang, Harbin, China; ^5^ Department of infectious disease prevention, Zhengzhou Centers for Disease Control and Prevention, Zhengzhou, China; ^6^ Department of HIV prevention, Tianjin Centers for Disease Control and Prevention, Tianjin, China; ^7^ Department of Epidemiology, China Medical University, Shenyang, China; ^8^ College of Pharmacy, University of Tennessee Health Science Center, Memphis, TN, United States; ^9^ National Center for Clinical Laboratories, Institute of Geriatric Medicine, Chinese Academy of Medical Sciences, Beijing Hospital/National Center of Gerontology, Beijing, China; ^10^ Beijing Engineering Research Center of Laboratory Medicine, Beijing Hospital, Beijing, China; ^11^ Clinical Research Academy, Peking University Shenzhen Hospital, Shenzhen, China

**Keywords:** PLWH, Sinovac CoronaVac, adverse events, neutralizing antibody, S-IgG antibody

## Abstract

**Background:**

People living with HIV (PLWH) are more vulnerable to SARS-CoV-2. However, evidence on the immunogenicity of coronavirus disease 2019 (COVID-19) vaccines in this population is insufficient. The objective of this study is to assess the immunogenicity and safety of the two-dose schedule of Sinovac CoronaVac for 6 months postvaccination in PLWH.

**Methods:**

We conducted a multicenter prospective cohort study among PLWH and HIV-negative adults in China. Participants who received two doses of CoronaVac prior to the recruitment were allocated into two groups and followed up for 6 months. The neutralizing antibodies (nAbs), immunoglobulin G against the receptor-binding domain of the spike protein (S-IgG), and gamma-interferon (IFN-γ) were measured to assess the associations among CoronaVac immunogenicity and related factors. Adverse reactions were collected to evaluate the safety profile of vaccination.

**Results:**

A total of 203 PLWH and 100 HIV-negative individuals were enrolled. A small portion of participants reported mild or moderate adverse reactions without serious adverse events. Median nAbs level in PLWH (31.96 IU/mL, IQR: 12.34-76.40) was lower than that in the control group (46.52 IU/mL, IQR: 29.08-77.30) at the 2-4 weeks postvaccination (*P*=0.002), and the same trend was presented for median S-IgG titer (37.09 vs. 60.02 IU/ml) (both *P <*0.05). The nAbs seroconversion rate in the PLWH group was also lower than in the control group (75.86% vs. 89.00%). After then, the immune responses reduced over time in term of only 23.04% of PLWH and 36.00% of HIV-negative individuals had a positive seroconversion for nAbs at 6-month. The multivariable generalized estimating equation analysis showed that PLWH with CD4+T count≥350 cells/µL presented higher immune response than PLWH with CD4+T count <350 cells/µL in terms of antibody seroconversion and titers. The immunogenicity did not differ in participants with low or high HIV viral load. The S-antigen specific IFN-γ immunity was generally stable and had a slow attenuation in both two groups for 6 months postvaccination.

**Conclusion:**

The Sinovac CoronaVac was generally safe and immunogenic in PLWH, but the immunity response was inferior and the antibodies vanished faster compared to HIV-negative individuals. This study suggested a shorter than 6-month interval of prime-boost vaccination for PLWH to ensure a better protection.

## Introduction

As of November 26, 2022, the coronavirus disease 2019 (COVID-19) pandemic caused by SARS-CoV-2 has resulted in more than 641 million cases and 6.6 million deaths globally (1). People living with HIV (PLWH) are more vulnerable to SARS-CoV-2 than the general population due to poorer immunity systems (2). World Health Organization (WHO) reported that about 38 million PLWH as of 2020 (3). COVID-19 vaccines are regarded as the most promising control method to curb the spread of SARS-CoV-2 (4). Therefore, the development of COVID-19 vaccines has been accelerated to prevent the spread of the virus and reduce the risk of severe illness. CoronaVac is the most widely utilized COVID-19 vaccine in China and has been introduced globally in more than 20 low-income and middle-income countries, such as Brazil, Chile, and Turkey (5–7). Studies showed that a two-dose CoronaVac regimen was effective in terms of an overall 67.7% (95% Confidence interval (CI), 35.9% to 83.7%) efficacy for the prevention of symptomatic COVID-19 and a 92% positivity of neutralizing antibodies (nAbs) among healthy adults (7–11).

Up to date, more than 20 clinical trials regarding COVID-19 vaccines in PLWH have been registered on ClinicalTrials.gov (12). In general, these studies have demonstrated favorable immunogenicity and efficacy of COVID-19 vaccines (13–17). However, few studies published data on the safety and immunogenicity of inactivated vaccines in PLWH. In a study that investigated 47 PLWH in Beijing, the antibody levels in PLWH have been maintained for at least three months, but PLWH with lower CD4+T-cell counts showed a poor antibody response to the inactivated vaccines (18). In studies conducted in Kunming (19), Chongqing (20), and Wuhan (21), PLWH displayed weaker immune responses to COVID-19 vaccination compared to HIV-negative participants. The immunoglobulin G against the receptor-binding domain of the spike protein (S-IgG) declined faster in the PLWH than those in the HIV-negative individuals, which indicated that a two-dose regimen may not be sufficient to provide persistent protection against SARS-CoV-2 among PLWH (22). A cross-sectional study demonstrated that poor immunological response was associated with impaired humoral response (23). The only prospective cohort study conducted among PLWH in Brazil reported that the S-IgG seroconversion rates and nAbs positivity differed among PLWH with higher or lower than 500 cells/μL of CD4+T count (24). However, this study failed to answer the long-time persistence of immunity of vaccination in terms of only a 69-days observation postvaccination. In summary, the above-mentioned studies were either cross-sectional or relatively short-period cohort studies that aimed to evaluate CoronaVac combined with a similar BBIBP-CorV vaccine. Hence, the previous findings might not be applicable to evaluate the performance of CoronaVac in PLWH as a reference.

Despite the fact that a national immunization campaign with CoronaVac had launched and immunized 88.01% populations in China up to 2022 (25), the immunization strategy against SARS-CoV-2 among PLWH falls behind. China adopted the same interval of prime-boost vaccine schedule as that of healthy people for PLWH at present, hence the interval between the 2nd and 3rd dose was about 6 months. However, lacking sufficient evidence about the long-term immunogenicity of the vaccine among PLWH lead to no systematic assessment of the immunological characteristics of the vaccine interval in PLWH. Besides, due to a great part of PLWH individuals with vaccine hesitancy presented low adherence to complete vaccination schedule, cohort studies aimed at evaluating the single CoronaVac vaccine among these special populations were rare in China (26). In order to address these knowledge gaps, we conducted a multicenter prospective cohort study to assess the safety and immunogenicity of a two-dose regimen of CoronaVac vaccine in PLWH with comparing to HIV-negative counterparts. Besides, this study also measured humoral responses to wild-type SARS-CoV-2 and evaluated the immune durability during the six months postvaccination. Considering that S-antigen specific gamma-interferon (IFN-γ) was induced by T-cell immunity, which was deemed to be associated with protection against SARS-CoV-2 and severe disease according to WHO guidelines and existing studies (27), S-antigen specific IFN-γ was also assessed in this study. The factors correlated with nAbs and S-IgG were further investigated. We hypothesized that the immunogenicity and safety of vaccination would differ between PLWH and HIV-negative individuals.

## Materials and methods

### Study design and objective

This prospective cohort study was launched in four Chinese metropolitan cities (Tianjin, Zhengzhou, Harbin, and Hohhot) between July 2021 and February 2022. PLWH and HIV-negative individuals aged 18 years and older were recruited for eligibility assessment through three methods: internet social software platform, snowball sampling through communities of PLWH, and respondent-driven sampling. All participants at the 2-4 weeks postvaccination with a two-dose schedule of CoronaVac were allocated into two groups in a ratio of 2:1 and followed for 6 months with 3 visits. This study aimed to assess the immunogenicity and safety of the inactivated COVID-19 vaccine (Sinovac CoronaVac), and the immune persistence of a two-dose regimen of CoronaVac for 6 months in PLWH.

### Participants

In this study, the inclusion criteria for participants included (1): aged 18–75 years (2); 2-4 weeks postvaccination with the second dose of Sinovac CoronaVac; and (3) being willing to participate in the study activities and signed written informed consent. The HIV infection was preliminarily identified by HIV rapid test kit in all participants. Besides, we re-identified the HIV serostatus for PLWH using Abbott ARCHITECT HIV Ag/Ab Combo assay at the study site. The exclusion criteria were (1): a presence of severe hearing loss, impaired vision, or intellectual disability observed by the interviewers (2); a history of SARS-CoV-2 infection which was defined by searching national medical records and self-reports (3). a history of vaccination with another COVID-19 vaccine instead of CoronaVac according to the national medical records; or (4) major psychiatric illness (schizophrenia or bipolar disorder) or other severe diseases which were not suitable for this study based on clinician’s assessment.

### Procedures

**Figure 1:**
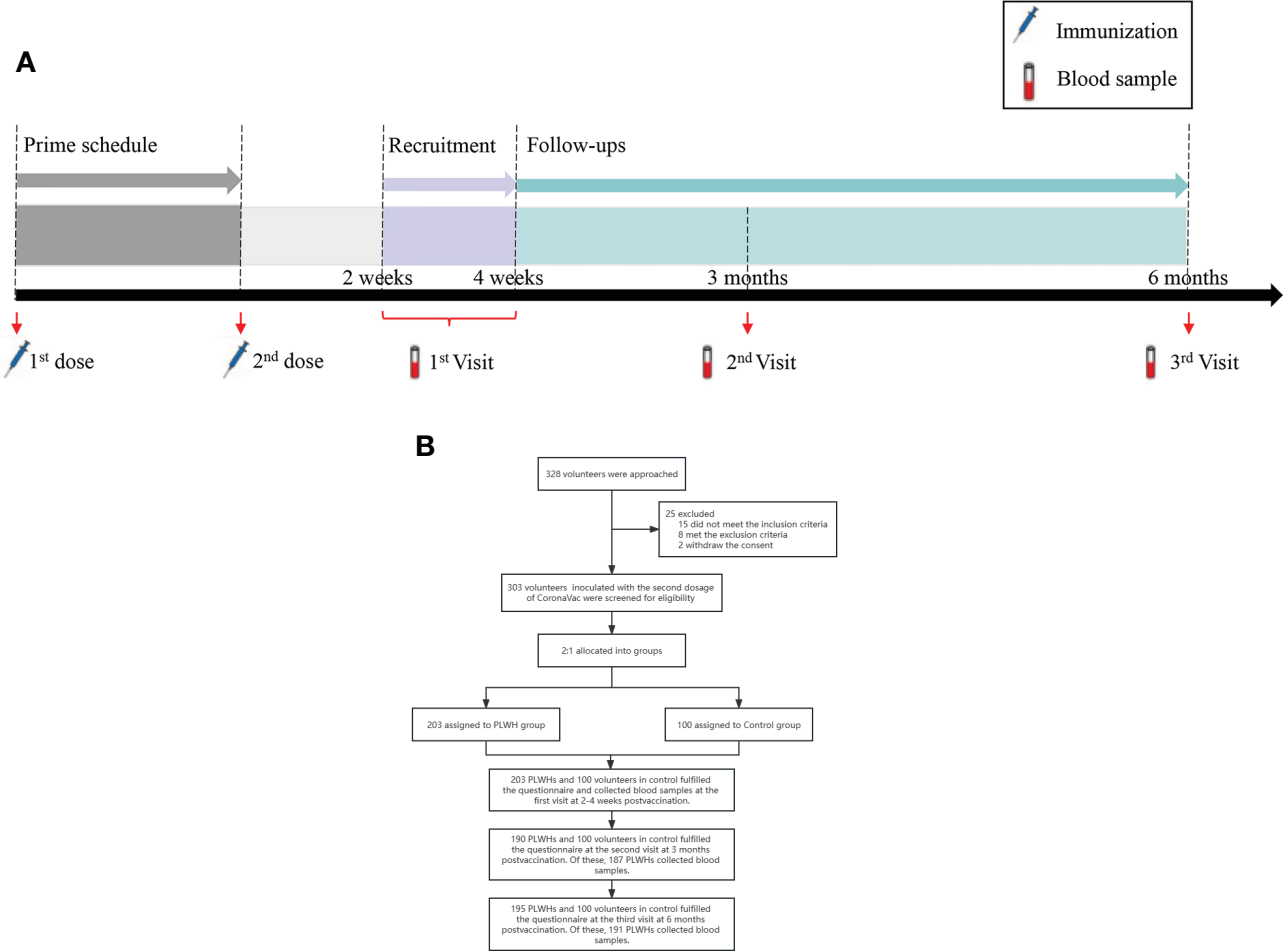
**(A)** The study procedures of immunization schedule and blood sample collection. **(B)** The flowchart of this study. PLWH, People living with HIV.

PLWH were recruited from four community-based organizations (CBOs) which collaborated with HIV clinical service providers and offered services to PLWH, one in each city. The recruitment advertisements were advocated through WeChat public accounts. Then the interested PLWH would contact project staffs *via* social media and be informed of the study purpose and procedure briefly. Potential PLWH were screened using inclusion criteria and a free HIV test through the HIV rapid test kit, and signed the informed consent after then. Eligible HIV-negative individuals were also invited to attend this study. All participants would be followed for 6 months with 3 visits at 2-4 weeks, 3 months, and 6 months postvaccination with the second dose of Sinovac CoronaVac (**Figure 1A**). While the participants were waiting for collecting blood samples at the site, the project staffs would instruct participants to complete a questionnaire through data management software (Jinshuju). Through the questionnaire, background data were collected, including age, gender, and presence of chronic conditions (i.e., hypertension, cancer, and kidney diseases, etc). COVID-19 vaccination information was extracted from the individual records of the national vaccination system upon participants’ consent. The questionnaire is anonymous with a unique 6-bit digital number for each participant, which link the questionnaire and the laboratory test results. A master list with identifiable information was saved in the principal investigator’s computer with password protection, only the principal investigator has access to it.

The safety profile of the CoronaVac vaccine was measured using the adverse events (AEs) and serious adverse events (SAEs) postvaccination. Self-reported AEs were collected through the questionnaire. The AEs mainly include local AEs (redness, pain, swelling, itching, skin rashes, and induration in the immunized arm) and systematic AEs (headache, fatigue, dizziness, joint pain, fever, nausea, vomiting, diarrhea, and others).

### Sample collection and laboratory procedures

A whole blood sample was collected from all participants by trained site nurses at each visit. The nAbs responses were tested through a qualitative competitive Chemiluminescence assay (CLIA) (Xiamen Wantai Biological Pharmacy Enterprise Co., Ltd., China). A cutoff value for nAbs was set as greater than 11.5 IU/ml. The S-IgG antibody titer was measured through an indirect CLIA method using the recombinant receptor-binding domain of the SARS-CoV-2 spike protein as antigen (Beijing Wantai Biological Pharmacy Enterprise Co., Ltd., China). The concentration of S-IgG ≥ 20 IU/ml was defined as S-IgG antibody positive. Moreover, the IFN-γ cell immune response assay kit (Xiamen Wantai Biological Pharmacy Enterprise Co., Ltd., China) which is designed to quantitatively detect the concentration of IFN-γ produced by SARS-CoV-2 specific T cells in whole blood samples was adopted. This kit can be used as an auxiliary diagnosis for the specific T-cell immune response of vaccinees. 500ul of heparin anticoagulant whole blood from participants was added into a test tube (T tube) that contains 10ug/mL specific SARS-CoV-2 S-antigen, a positive control tube (P tube) contains Phorbol-12-myristate-13-acetate (PMA), and a negative control tube (N tube). Tubes were cultured at 37°C for 20-24h. After then, the tubes were centrifuged at 3000 g for 10 min, and the plasma collected from upper layer was assessed for IFN-γ level using a quantitative CLIA method. The positive for S-antigen specific IFN-γ level was defined as the value of T tube minus N tube greater than 30 pg/mL.

Additionally, we adopted HIV quantitative assay (Zhuhai Livzon Diagnostics Inc., Zhuhai, China) to detect the HIV viral load for PLWH. An HIV viral load positive was set as more than 18 copies/mL. A flow cytometry test (BD Biosciences, San Jose, CA, USA) was also performed to count CD4+T cells based on the China National Guideline for Detection of HIV/AIDS (version 2021) (28). The tests of the above samples were performed in duplicate and anonymous, followed by a double-check and unblinding at the end.

### Sample size planning

The foremost objective of this study is to assess the immunogenicity of the CoronaVac vaccine among PLWH compared with HIV-negative adults up to 6 months upon completing the two-dose vaccination. Immunogenicity was defined as the percentage of participants achieving a seroconversion response of nAbs. Previous studies showed that seroconversion for nAbs ranged from 80% to 90% among healthy adults (9–11) and was 71% among PLWH in the Brazil study (24). We assumed that a seroconversion rate of nAbs is 70% in PLWH and 87% in healthy adults. Therefore, the sample size of at least 160 PLWH and 80 HIV-negative participants with an allocation ratio of 2:1 would provide at least 90% of power to detect a difference beyond the pre-specified non-inferiority margin of -10% (α = 0.05, β = 0.10). A total of 300 participants were finally required after considering the 20% rate of loss to follow-up. The sample size was calculated using Power Analysis and Sample Size software (version 15.0.5).

### Statistics analysis

Descriptive statistics were used to present the background characteristics. We adopted Fisher’s exact or χ2 for categorical data to inspect the difference in the AEs and antibody seroconversion related to CoronaVac vaccination between PLWH and HIV-negative participants. The nAbs and S-IgG levels were described using medians and inter-quartile ranges (IQR). The antibody level values were tested using Mann–Whitney tests. We categorized variables of HIV viral load (0-18 copies/mL, ≥19 copies/mL), and CD4+T count (<350 cells/µL, ≥350 cells/µL) into subgroups based on their values. Furthermore, we performed a generalized estimating equation (GEE) model to assess the longitudinal nature of immunogenicity assessments which were based on the effect of HIV infection and CD4+T cell counts on the concentrations of S-IgG and nAbs titer at baseline, 3 month and 6 month adjusted for age and gender. Missing data were not included in the analysis. All statistical analyses were performed using SAS software (version 9.1), with a two-tailed *P*< 0.05 considered statistically significant. The pictures were plotted using GraphPad Prism (version 9.0.0). STROBE guidelines were followed for the presentation of all study results.

## Results

### Participants

A total of 328 participants aged between 18 and 75 years old in Tianjin, Zhengzhou, Harbin, and Hohhot were approached for participation between July 2021 and February 2022. Of them, 203 PLWH and 100 healthy individuals were finally enrolled in the study. Twenty-five participants were excluded due to logistical reasons or not eligible or self-willingness (**Figure 1B**). All participants who have met the procedure requirements completed the first visit by fulfilling the questionnaire and collecting blood samples. At the second visit, 190 PLWHs and 100 volunteers in control fulfilled the questionnaire. Of which, only 187 PLWHs who donated blood samples were included in the immunogenic analysis, 3 individuals could not attend the site due to the restriction from epidemic. Similarly, 191 of 195 PLWHs and 100 controls collected the blood samples at the third visit. The average age for PLWH was 35 (IQR: 28-44) years old, and male participants accounted for the majority of the cohort (196/203, 96.55%). Over half of the participants were single (113/203, 55.67%) and with a college and above education level (115/203, 56.65%). Most PLWH received antiretroviral therapy (ART) (196/203, 96.56%) and had an undetectable viral load (166/203, 81.77%). The median HIV viral load was undetectable and CD4+T cell counts were 546.00 cells/µL (IQR: 361.50-675.75), respectively. Compared to the control group, PLWH had a higher proportion of males and were younger. In general, comorbidity rates in PLWH were higher than those in the control group at the baseline (14.8% v.s. 5.0%, *P*=0.012). Hypertension was the most common comorbidity in PLWH (3.45%), as well as in the control group (2.00%, **Table 1**). A significant difference was found in the occurrence of any comorbidities between PLWH and the control group (*P*=0.012), but not in any specific comorbidity.

**Figure 2:**
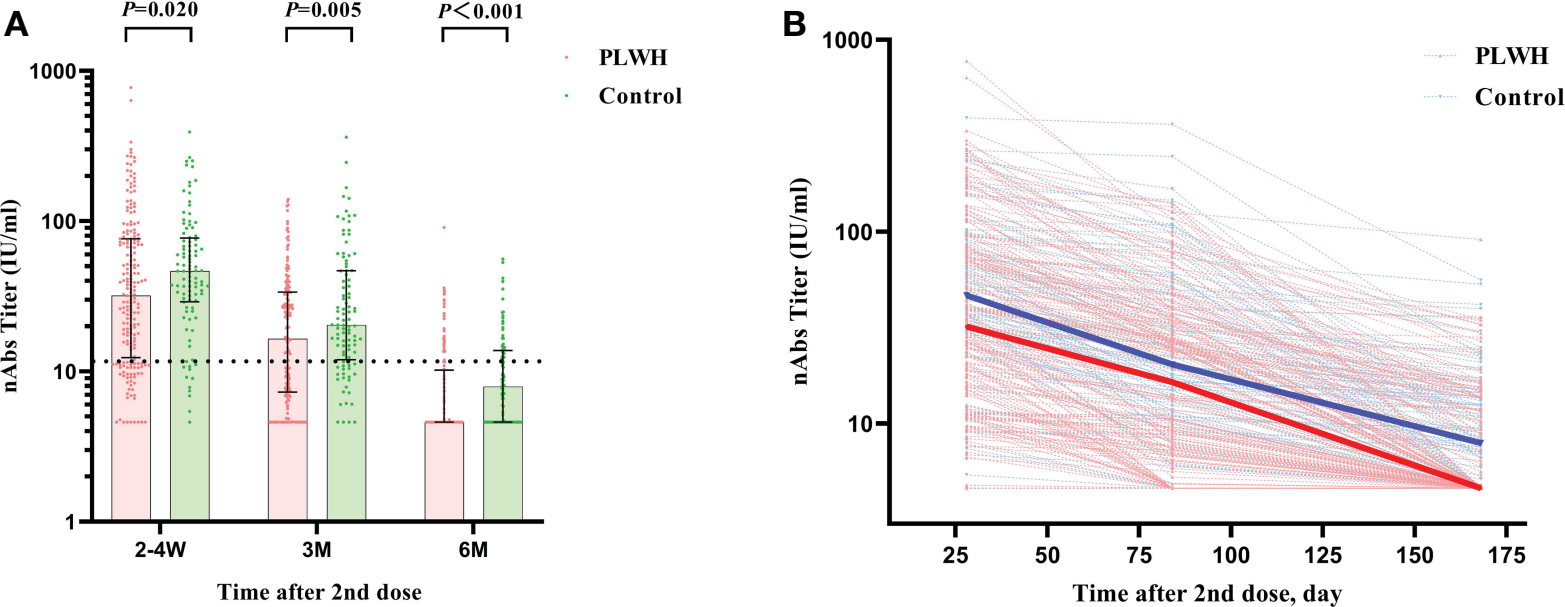
**(A)** The comparison of nAbs titers between PLWH and HIV-negative individuals at 2-4 weeks, 3 months, and 6 months postvaccination by Mann–Whitney tests. The vertical dotted line represents the cut-off value for antibody titers. PLWH, People living with HIV; nAbs, neutralizing antibodies. **(B)** The reduction curves for nAbs titers between PLWH and HIV-negative individuals over time. The solid lines represent the median curve for nAbs titers between PLWH and HIV-negative individuals over time.

**Table 1:** The comparison of background characteristics for HIV-negative individuals and PLWH who had been recruited in the study with prime immunization of CoronaVac vaccines by Fisher’s exact or χ2 test.

Variable	PLWH (n=203)	HIV-negative (n=100)	*P* value
Age (years)	35 (28–44)	40.5 (28.3-51.0)	<0.001
18-45	162 (79.80%)	57 (57.00%)	
46-59	38 (18.72%)	40 (40.00%)	
60-75	3 (1.48%)	3 (3.00%)	
Gender			<0.001
Male	196 (96.55%)	83 (83.00%)	
Female	7 (3.45%)	17 (17.00%)	
CD4+T cell count (per uL)			
<350	47/199(23.62%)	N.A.	N.A.
≥350	152/199(76.38%)	N.A.	N.A.
Plasma HIV viral load (copies/mL)			
<18	166 (81.77%)	N.A.	N.A.
≥18	37 (18.23%)	N.A.	N.A.
ART regimens			
TDF + 3TC + EFV	113 (55.67%)	N.A.	N.A.
TDF + 3TC + NVP	3 (1.48%)	N.A.	N.A.
TDF + 3TC + LPV/r	11 (5.42%)	N.A.	N.A.
AZT + 3TC + LPV/r	3 (1.48%)	N.A.	N.A.
AZT + 3TC + NVP	2 (0.99%)	N.A.	N.A.
AZT + 3TC + EFV	4 (1.97%)	N.A.	N.A.
Others*	60 (29.56%)	N.A.	N.A.
Not on ART	7 (3.45%)	N.A.	N.A.
Interval between two doses (days)			0.026
<21	9 (4.40%)	5 (5.00%)	
21-28	131 (64.50%)	49 (49.00%)	
>28	63 (31.00%)	46 (46.00%)	
Comorbidities			
Any comorbidity	30 (14.78%)	5 (5.00%)	0.012
Hypertension	7 (3.45%)	2 (2.00%)	0.723
Hyperlipoidemia	3 (1.48%)	0 (0.00%)	0.553
Diabetes	2 (0.99%)	0 (0.00%)	>0.999
Thrombocytopenia	4 (1.97%)	0 (0.00%)	0.306
Mental illness	3 (1.48%)	0 (0.00%)	0.553
Chronic kidney disease	4 (1.97%)	0 (0.00%)	0.306
Others	9 (4.43%)	3 (3.00%)	0.757
Career			0.036
Retired	72 (35.50%)	48 (48.00%)	
Full time	131 (64.50%)	52 (52.00%)	
Smoking			0.020
No	141 (69.50%)	82 (82.00%)	
Yes	62 (30.54%)	18 (18.00%)	
Alcohol intake			0.049
No	150 (73.90%)	84 (84.00%)	
Yes	53 (26.10%)	16 (16.00%)	

Data are presented as median (IQR) or n/N (%) or n (%).

PLWH, people live with HIV; N.A, not applicable; ART, antiretroviral therapy; TDF, tenofovir disoproxil fumarate; 3TC, lamivudine; EFV, efavirenz; NVP, nevirapine; LPV/r, lopinavir/ritonavir; AZT, Zidovudine.

*Others contains unclear of the regimen and other regimens which were not displayed.

### Safety

A total of 18 (8.87%) of 203 participants in PLWH group and 5 (5.00%) of 100 participants in the healthy control group reported at least one adverse reaction at 2-4 weeks postvaccination (**Table 2**). During the 3 months and 6 months visits, only 2 (1.05%) and 1 (1.05%) adverse reactions occurred in PLWH, respectively. There were no cases documented in the control group. Most reactions were mild or moderate and no reports of any SAEs. Though a higher proportion of total adverse reactions in the PLWH group was noticed, no significant difference was found between the PLWH and the HIV-negative individuals at three times visits (all *P*>0.05).

**Table 2:** The comparison of adverse reactions of CoronaVac prime vaccination schedule in participants during the whole following period by Fisher’s exact or χ2 test.

Adverse reaction	PLWH	HIV-negative	*P* value
2-4 weeks	n=203	n=100	
Total adverse reactions	18 (8.87%)	5 (5.00%)	0.232
Injection site adverse reactions	7 (3.45%)	3 (3.00%)	>0.999
Pain	5 (2.46%)	2 (2.00%)	>0.999
Induration	1 (0.49%)	/	>0.999
Redness	2 (0.99%)	/	0.330
Rash	/	1 (1.00%)	>0.999
Itch	1 (0.49%)	/	>0.999
Systemic adverse reactions	14 (6.90%)	2 (2.00%)	0.073
Allergy	2 (0.99%)	1 (1.00%)	>0.999
Fatigue	9 (4.43%)	/	0.033
Diarrhea	3 (1.48%)	/	0.553
Skin and Mucosal tissue disorders	2 (0.99%)	/	>0.999
Asitia	2 (0.99%)	1 (1.00%)	>0.999
Vomit	1 (0.49%)	/	>0.999
Nausea	2 (0.99%)	/	>0.999
Myalgia/Arthralgia	1 (0.49%)	/	>0.999
Headache	1 (0.49%)	/	>0.999
Cough	1 (0.49%)	/	>0.999
Fever	1 (0.49%)	/	>0.999
3 months	n=190	n=100	
Total adverse reactions	2 (1.05%)	/	0.547
Injection site adverse reactions	1 (0.53%)	/	>0.999
Rash	1 (0.53%)	/	>0.999
Systemic adverse reactions	2 (1.05%)	/	0.547
Fatigue	1 (0.53%)	/	>0.999
Nausea	1 (0.53%)	/	>0.999
6 months	n=195	n=100	
Total adverse reactions	1 (1.05%)	/	>0.999
Systemic adverse reactions	1 (1.05%)	/	>0.999
Allergy	1 (1.05%)	/	>0.999

Data are n (%), representing the total number of participants who had adverse reactions (ie, adverse events related to vaccination).

PLWH, people live with HIV.

Generally, the CoronaVac vaccine elicited more systemic AEs in PLWH than did the healthy control at the 2-4 weeks postvaccination numerically without significant difference (6.90% v.s. 2.00%). Similarly, there was no difference in the injection site AEs between the two groups. After then, the incidences of AEs reduced over time in both two groups. Only 1.05% systemic AEs and 0.53% local AEs occurred at 3-months visit in the PLWH, and no more local AEs were reported at the 6-months visit.

### Immunogenicity

In general, the two-dose vaccination of Sinovac CoronaVac elicited significant immune responses in both the PLWH group and the control group as measured by nAbs and S-IgG. The nAbs titers against SARS-CoV-2 at 2-4 weeks postvaccination showed the highest value among the 3-time visits and the values showed a decreased trend in both the PLWH group and the control group after then (**Table 3**). The median titer of nAbs for the PLWH group and the control group was 31.96 IU/ml (IQR: 12.34-76.40) and 46.52 IU/ml (IQR: 29.08-77.30), respectively. The seroconversion rates for nAbs were 75.86% and 89.00% at baseline in the PLWH group and the control group. This result indicated that part of the participants received protection through vaccination, but the seroconversion rate in the PLWH group was significantly lower than the one in the control group (*P*=0.007). At the second visit, only around half of PLWH had a seroconversion for nAbs (57.75%), compared to 77.00% in the control group. The median nAbs titer was reduced to 16.44 IU/ml (IQR: 7.29-33.74) and 20.34 IU/ml (IQR: 11.95-46.88) in the PLWH group and control group, respectively. At 6 months, the median titers in both groups were lower than the cutoff value of 11.5IU/ml (**Figure 2A**). Only 23.04% of PLWH and 36.00% of HIV-negative individuals had a seroconversion at 6-months visit. In addition, we also observed a sharper reducing trend in PLWH than the ones in the control group (**Figure 2B**).

**Table 3:** The comparison of immunogenicity profiles of CoronoVac two dose schedule in participants during 6 months postvaccination by Fisher’s exact or χ2 test.

	HIV-positive (n=203)	HIV-negative (n=100)	*P*-value
2-4 weeks			
**nAbs positivity**	154/203 (75.86%)	89/100 (89.00%)	0.007
**nAbs titer**	31.96 (12.34-76.40)	46.52 (29.08-77.30)	0.020
**S-IgG Seroconversion**	152/203 (74.88%)	88/100 (88.00%)	0.008
**S-IgG titer**	37.09 (19.32-81.73)	60.02 (40.79-79.52)	<0.001
**IFN-γ**	141/203 (69.46%)	74/99 (74.75%)	0.341
3 Months			
**nAbs positivity**	108/187 (57.75%)	77/100 (77.00%)	0.001
**nAbs titer**	16.44 (7.29-33.74)	20.34 (11.95-46.88)	0.005
**S-IgG Seroconversion**	91/187 (48.66%)	64/100 (64.00%)	0.013
**S-IgG titer**	18.63 (9.22-30.94)	25.57 (15.23-44.10)	<0.001
**IFN-γ**	109/186 (58.60%)	62/100 (62.00%)	0.576
6 Months			
**nAbs positivity**	44/191 (23.04%)	36/100 (36.00%)	0.019
**nAbs titer**	4.60 (4.60-10.20)	7.91 (4.60-13.77)	<0.001
**S-IgG Seroconversion**	59/191 (30.89%)	41/100 (41.00%)	0.085
**S-IgG titer**	8.42 (3.48-21.36)	13.65 (5.63-24.89)	0.012
**IFN-γ**	64/127 (50.39%)	52/100 (52.50%)	0.810

Data are presented as median (IQR) or n/N (%). The S-IgG seroconversion, which was defined as immune response from a negative to a positive status, is characterized as a value more than 20 IU/mL. The nAbs positivity is defined as a value greater than 11.5 IU/mL. The IFN-γ positivity is defined as a value greater than 30 pg/mL.

PLWH, people live with HIV; nAbs, neutralizing antibody.

**Figure 3:**
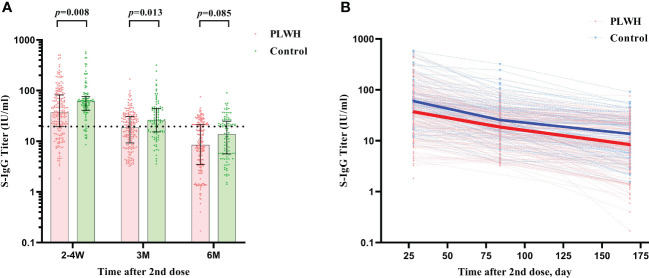
**(A)** The comparison of S-IgG antibody titers between PLWH and HIV-negative individuals at 2-4 weeks, 3 months, and 6 months postvaccination by Mann–Whitney tests. The vertical dotted line represents the cut-off value for antibody titers. PLWH, People living with HIV. **(B)** The reduction curves for S-IgG antibody titers between PLWH and HIV-negative individuals over time. The solid lines represent the median curve for S-IgG antibody titers between PLWH and HIV-negative individuals over time.

**Figure 4:**
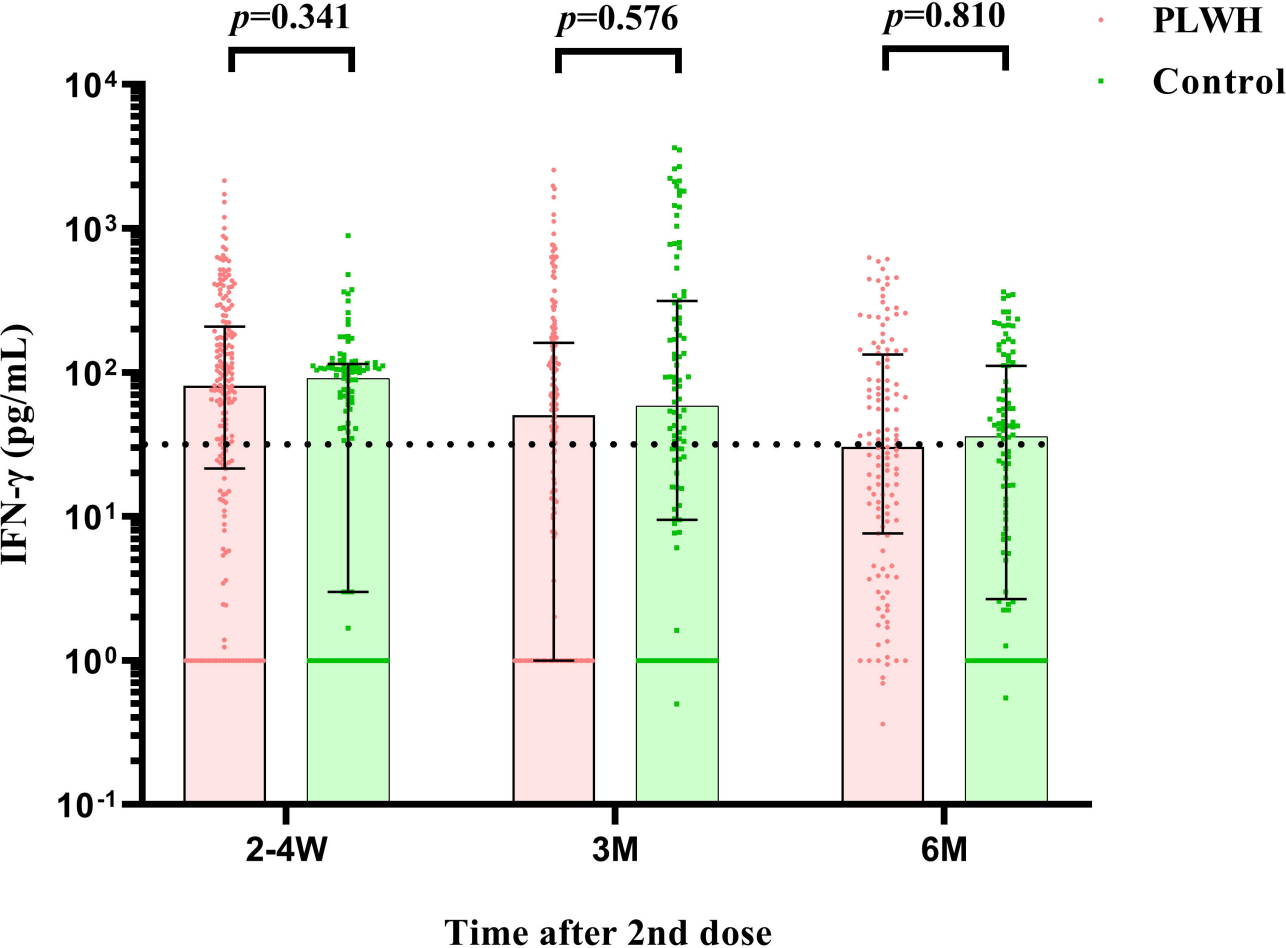
The comparison of IFN-γ cell counts between PLWH and HIV-negative individuals at 2-4 weeks, 3 months, and 6 months postvaccination by Mann–Whitney tests. PLWH, People living with HIV.

Similar results were found for the S-IgG immunity indicator. At the first visit, HIV-negative participants showed a significantly higher S-IgG antibody level of 60.02 IU/ml (IQR: 40.79-79.52) than PLWH, which had a titer of 37.09 IU/ml (IQR: 19.32-81.73) (*P*<0.001). The seroconversion rate for S-IgG was 74.88% and 88.00% in PLWH and control, respectively (**Figures 3A, B**). After then, the S-IgG antibody decreased from 18.63 IU/ml to 8.42 IU/ml in the PLWH group at the last 2 visits, while the S-IgG antibody was reduced from 25.57 IU/ml to 13.65 IU/ml in the control group. At the same time, the seroconversion rate was only 30.89% and 41.00% for PLWH and the control, respectively (**Table 3**). Regarding the IFN-γ, a similar postvaccination trend was observed in both groups at 3 visits with no significant difference (**Figure 4**).

Factors associated with immunogenicity indicator levels in all participants was preliminarily evaluated through univariate GEE model (**Supplementary Table S1**). The results showed that, compared with PLWH with low CD4+T counts, those with high CD4+T counts and HIV negative individuals had higher seroconversion rates for nAbs and S-IgG. After then, the multivariable GEE model was adopted to assess the effect of the above significant variables and variables with *P* < 0.1 on S-IgG and nAbs activity with adjusting for age and gender in all participants (**Table 4**). HIV-negative individuals and PLWH with a higher than 350 cells/µL CD4+T count reported an increase in nAbs activity and S-IgG seroconversion across 3 visits compared to PLWH with a lower than 350 cells/µL CD4+T count. Further, the HIV viral load level did not show a significant association with nAbs positivity and S-IgG seroconversion among PLWH after adjusting confounding variables.

**Table 4:** The multivariable generalized estimating equation analysis of factors associated with S-IgG and nAbs postvaccination adjusted with gender and age during three visits in all participants.

	nAbs positivity		S-IgG seroconversion	
	aRR (95%CI)	*P*-value	aRR (95%CI)	*P*-value
CD4+T count				
PLWH, CD4 count<350cells per uL	Ref	/	Ref	/
PLWH, CD4 count ≥350 cells per uL	2.243 (1.420-3.541)	0.001	1.995 (1.184-3.360)	0.009
HIV-negative individuals	3.152 (1.922-5.169)	<0.001	2.575 (1.483-4.471)	0.001
HIV viral load				
PLWH, Viral load <18 copies/mL	Ref	/	Ref	/
PLWH, Viral load ≥18 copies/mL	0.891 (0.528-1.503)	0.665	1.342 (0.722-2.497)	0.352
HIV-negative individuals	1.708 (1.180-2.472)	0.005	1.581 (1.076-2.322)	0.020

PLWH, people live with HIV; nAbs, neutralizing antibody; Ref, reference group; aRR, adjusted risk ratio; 95%CI, 95% confidence interval.

## Discussion

In this multicenter prospective cohort study, we investigated the safety and immunogenicity profiles of a two-dosage regimen of the CoronaVac vaccine up to 6 months postvaccination among PLWH. We found the CoronaVac vaccine was well-tolerance and acceptable among PLWH without causing any SAEs. In general, the CoronaVac vaccine elicited robust immune responses to the SARS-CoV-2 among PLWH during the 6-month period. Moreover, the immunogenicity of the vaccine was significantly lower among PLWH compared to HIV-negative individuals, as measured the nAbs and S-IgG, which indicated an earlier boost dose may be needed for PLWH. In particular, S-antigen specific IFN-γ induced by T-cell immune response showed no association with HIV status and maintained stable for 6 months postvaccination with a slow attenuation. The results of our study added evidences to the characteristics and differences in immune response between PLWH and HIV-negative individuals, which is essential in formulating specific vaccination guidelines for PLWH.

The PLWH presented similar rates of AEs compared to HIV-negative individuals except for fatigue, which showed a higher rate in PLWH than in the control group at 2-4 weeks postvaccination. This phenomenon may be associated with the dysfunction of the immune system among PLWH. All reported AEs were mild or self-limited, which was consistent with other studies that investigated inactivated vaccines among PLWH (18, 20, 24).

In the aspect of the immunogenicity profile, the CoronaVac vaccine elicited a high level of nAbs and S-IgG antibodies to the antigens among PLWH. The levels and seroconversion rates of these two antibodies peaked at 14-28 days postvaccination, which was consistent with other studies that investigated COVID-19 vaccines in HIV-negative individuals or PLWH (11, 29, 30). However, contrary to another study with long follow-up period, the peak immunogenicity of the inactivated COVID-19 vaccine was not delayed in PLWH compared to control in our research (31). The peak levels and seroconversion rates of the S-IgG and nAbs among PLWH were significantly lower than the control group at all visits (*P*<0.05). This may be caused by the dysfunction of the immune system among PLWH, which was in consistent with other studies. We also noticed a faster decline in S-IgG among PLWH compared to HIV-negative individuals. At the last visit, the S-IgG titer declined to the bottom and failed to confer robust immunity among PLWH, whereas the S-IgG titer remained stable among HIV-negative individuals. Similar results were obtained for nAbs. The patterns of lower peak levels and faster decline trend of immune responses in PLWH could possibly be explained by the lower CD4+T cell compared with healthy individuals, which was associated with impaired cellular immunity and B-cell dysfunction in PLWH. The findings suggested that PLWH may need an additional booster dose on top of the two doses. In face of continuous epidemics, an additional dose of COVID-19 vaccine was proved to be effective and induced significant higher antibody levels compared with the second dose (32–34). Since PLWH have an increased risk of breakthrough infections compared to the general population, a booster dose to PLWH would be an alternative method to sustain sufficient protection. Though China is promoting a prime-boost schedule, a high proportion of PLWH failed to receive a booster dose due to vaccine hesitancy (35). Considering that the Chinese government has recently published novel guidance regarding a shorter interval of prime-boost schedule (3 months) among elderly than younger adults (6 months), this finding will provide strong evidence to formulate specific vaccination guidance in PLWH (36). The study findings on the safety and immunogenicity of COVID-19 vaccine have implications in increasing awareness of COVID-19 vaccination and contribute to improving the coverage of vaccination.

Since the S-antigen specific IFN-γ among vaccinees was mainly secreted by activated Th1 CD4+ cells and CD8+ cytotoxic T lymphocyte effector T cells once antigen-specific immunity develops, the IFN-γ induced by T-cell immune response was also assessed in our study. Our results showed that the level of cellular immune response was not associated with HIV status, but maintained stable for 6 months upon vaccination with a slow attenuation in two groups. The literature has proved that the T-cell immunity could significantly reduce the incidence of severe diseases and deaths of COVID-19, and the cross-reactivity between the T-cell response and omicron mutants was high (37, 38), which strengthened the importance for detection of S-antigen specific IFN-γ. Although the protection through vaccination may be inferior in PLWH than in HIV-negative individuals, vaccination still developed sufficient protection against severe diseases induced by omicron variants in PLWH (39). Future studies are warranted to determine further relationship between vaccine-induced S-antigen specific IFN-γ level and protection against COVID-19, and gather evidences to clarify the role of specific IFN-γ cellular response through different prime-boost regimens.

We noticed that the PLWH with less than 350 cells/µL CD4+T counts had lower immunity responses to vaccination than those with higher CD4+T cell counts, which was possible because that PLWH with well-controlled HIV disease under ART had abundant CD4+T counts to provide better-functioning immune system. Subgroup analysis showed that PLWH with higher CD4+ T counts and undetectable HIV viral loads did not have significantly lower neutralizing activity and S-IgG antibody levels compared to HIV-negative individuals. This result is in line with previous studies (24). However, PLWH with lower CD4+T counts had lower neutralizing activity levels, which indicated PLWH with lower CD4+T counts may be more vulnerable to SARS-CoV-2 than PLWH with higher CD4+T counts.

This study has several strengths. First of all, the participants were prospectively followed up for 6 months, which was longer than other previous studies in China (18, 19, 22). The follow-up period is important and could provide compelling evidence to identify an optimal vaccination schedule for PLWH. Second, the sample size of PLWH was larger than those in previous studies (31), which allowed us to exclude the effect of confounding variables by using a multivariable model and guaranteed the reliability of results. Third, our study was the first and only cohort study that assessed the safety and immunogenicity of the single CoronaVac vaccine in PLWH at present. Previous studies have investigated the combination of CoronaVac and other inactivated vaccines in mainland China (20, 22). Fourth, humoral and cellular immune response analyses were included in this study, which helps to fill the knowledge gap regarding characteristics of cellular immune response in PLWH, especially in the prevention of severe clinical outcomes.

Despite the advantages, this prospective cohort study also has a few limitations. First, gender and age distributions varied between the two groups. To reduce its impact on the study results, we adjusted for gender and age in the multivariable GEE model. Second, recall bias, which was hardly avoided in prospective cohort study might potentially affect the accuracy of the information collected through a questionnaire, especially for the AEs information. Third, since the antibody activity was quantified only to wild strain, we were unable to assess whether the vaccine could induce cross-protection against novel mutant strains. Future studies are needed to investigate the protection against other mutant strains and measure immune responses to the COVID-19 vaccine in a longer period using these immunogenicity indicators among PLWH.

The findings of our study showed that the inactivated Sinovac CoronaVac vaccine was safe and immunogenic in PLWH. However, the immune response among PLWH was generally inferior to those in HIV-negative individuals and the antibody against SARS-CoV-2 decreased rapidly over time in PLWH, indicating the vaccine was not capable of providing ideal protection for 6 months. A specific shorter interval strategy between the prime two dosages and the boost dosage for PLWH should be considered, especially for PLWH with lower CD4+T cell counts to ensure sufficient protection against SARS-CoV-2. The S-antigen specific IFN-γ immunity was high and had a slow attenuation trend even 6 months postvaccination, suggesting that the COVID-19 vaccine has a preventive effect on severe disease of COVID-19 in PLWH and the healthy people.

## Data availability statement

The raw data supporting the conclusions of this article will be made available by the authors, without undue reservation.

## Ethics statement

The studies involving human participants were reviewed and approved by the institutional review board of Peking University Shenzhen Hospital, and done in accordance with the Declaration of Helsinki, Good Clinical Practice, and Chinese regulatory requirements (No. 2021-041). Written informed consents were obtained from participants or their guardians prior to enrolment. The patients/participants provided their written informed consent to participate in this study.

## Author contributions

JX designed this cohort study and was responsible for the data analysis. YW supervised data analysis, interpretation of data, and original drafting of the manuscript. XL, MS and XZ contributed to data curation and performed statistical analysis. YQ and YH trained and supervised field staff and coordinated field logistics. LW, SL and MY reviewed the study protocol, oversaw the conduct of the field work. YY was responsible for the laboratory tests and oversaw quality control of data collection. All authors contributed to the article and approved the submitted version.
