# Supplementary material for: The safety and immunogenicity of a two-dose schedule of CoronaVac, and the immune persistence of vaccination for six months, in people living with HIV: A multicenter prospective cohort study
Source: Front Immunol. 2023 Mar 13;14:1129651. doi: 10.3389/fimmu.2023.1129651 (PMC10040764; doi:10.3389/fimmu.2023.1129651)
Supplement: Supplementary file 1 [file Table_1.docx]

Supplementary Material

The safety and immunogenicity of a two-dose schedule of CoronaVac, and the immune persistence of vaccination for six months, in people living with HIV: a multicenter prospective cohort study

**Authors: Yuxiao Wang^1#^, Ying Qiao^2#^, Yuqi Huo ^3#^, Li Wang ^4^, Shijie Liang^5^, Maohe Yu^6^, Xinquan Lan^7^, Moxin Song^7^, Xiangjun Zhang ^8^, Ying Yan^9,10^*, Junjie Xu^11^***

*** Correspondence:** Co-Corresponding Author：

Junjie Xu; Email: xjjcmu@163.com;

Ying Yan; Email: [yyan511@nccl.org.cn](mailto:yyan511@nccl.org.cn);

# Supplementary Tables

Supplementary Table S1. Factors associated with SIgG and neutralizing antibody positivity postvaccination during three times visits in all participants

|  | **nAbs positivity** | | **S-IgG seroconversion** | |
| --- | --- | --- | --- | --- |
|  | RR(95%CI) | *P*-value | RR(95%CI) | *P*-value |
| **HIV Status** |  |  |  |  |
| PLWH, CD4 count<350cells per uL | Ref | / | Ref | / |
| PLWH, CD4 count ≥350 cells per uL | 2.182 (1.393-3.418) | 0.001 | 1.992 (1.186-3.344) | 0.009 |
| HIV-negative individuals | 3.202 (1.993-5.145) | ＜0.001 | 2.774 (1.611-4.778) | ＜0.001 |
| Viral load <18 copies/mL | Ref | / | Ref | / |
| Viral load ≥18 copies/mL | 0.907 (0.542-1.519) | 0.711 | 1.343 (0.724-2.492) | 0.350 |
| HIV-negative individuals | 1.764 (1.248-2.493) | 0.001 | 1.696 (1.176-2.448) | 0.005 |
| **The interval between the two doses** |  |  |  |  |
| ＜21 days | Ref | / | Ref | / |
| 21-28 days | 1.126 (0.538-2.356) | 0.753 | 1.322 (0.501-3.489) | 0.572 |
| ＞28 days | 1.089 (0.532-2.227) | 0.460 | 1.161 (0.431-3.127) | 0.768 |
| **Comorbidities** No | Ref | / | Ref | / |
| Yes | 1.151 (0.748-1.773) | 0.522 | 0.794 (0.463-1.360) | 0.400 |
| **Smoking** No | Ref | / | Ref | / |
| Yes | 0.876 (0.614-1.251) | 0.467 | 1.126 (0.752-1.686) | 0.563 |
| **Alcohol intake*** No | Ref | / | Ref | / |
| Yes | 1.038 (0.725-1.486) | 0.840 | 1.344 (0.890-2.030) | 0.160 |
| **Career**  Retired | Ref | / | Ref | / |
| Full time | 1.091 (0.787-1.512) | 0.602 | 1.111 (0.787-1.567) | 0.550 |
| **Gender** Female | Ref | / | Ref | / |
| Male | 0.571 (0.324-1.008) | 0.053 | 0.414 (0.231-0.744) | 0.003 |
| **Age**  18-45 years old | Ref | / | Ref | / |
| 46-60 years old | 1.203 (0.830-1.742) | 0.329 | 1.134 (0.775-1.659) | 0.518 |
| 60-75 years old | 0.398 (0.157-1.006) | 0.051 | 0.688 (0.259-1.828) | 0.453 |

Abbreviations: PLWH=people live with HIV; nAbs=neutralising antibody; Ref: reference group; RR= risk ratio.

*represents the participants whether had the history of alcohol intake.
